# The MIntAct project—IntAct as a common curation platform for 11 molecular interaction databases

**DOI:** 10.1093/nar/gkt1115

**Published:** 2013-11-13

**Authors:** Sandra Orchard, Mais Ammari, Bruno Aranda, Lionel Breuza, Leonardo Briganti, Fiona Broackes-Carter, Nancy H. Campbell, Gayatri Chavali, Carol Chen, Noemi del-Toro, Margaret Duesbury, Marine Dumousseau, Eugenia Galeota, Ursula Hinz, Marta Iannuccelli, Sruthi Jagannathan, Rafael Jimenez, Jyoti Khadake, Astrid Lagreid, Luana Licata, Ruth C. Lovering, Birgit Meldal, Anna N. Melidoni, Mila Milagros, Daniele Peluso, Livia Perfetto, Pablo Porras, Arathi Raghunath, Sylvie Ricard-Blum, Bernd Roechert, Andre Stutz, Michael Tognolli, Kim van Roey, Gianni Cesareni, Henning Hermjakob

**Affiliations:** ^1^European Molecular Biology Laboratory, European Bioinformatics Institute (EMBL-EBI), Wellcome Trust Genome Campus, Hinxton, Cambridge CB10 1SD, UK, ^2^School of Animal and Comparative Biomedical Sciences, The University of Arizona, Tucson, AZ 85721-0036, USA, ^3^Swiss-Prot Group, SIB Swiss Institute of Bioinformatics, CMU, 1 Rue Michel-Servet, CH-1211 Geneva 4, Switzerland, ^4^Department of Biology, University of Rome Tor Vergata, Rome 00133, Italy, ^5^Ontario Cancer Institute, the Campbell Family Institute for Cancer Research, and Techna Institute, University Health Network, Toronto, Ontario M5G 0A3, Canada, ^6^Cardiovascular Gene Annotation Initiative, Centre for Cardiovascular Genetics, Institute of Cardiovascular Science, University College London, London WC1E 6BT, UK, ^7^Centre for Microbial Diseases and Immunity Research, University of British Columbia, Vancouver, British Columbia V6T 1Z4 Canada, ^8^Mechanobiology Institute, National University of Singapore, T-Lab #05-01, 5A Engineering Drive 1, Singapore 117411, Singapore, ^9^Department of Cancer Research and Molecular Medicine, Norwegian University of Science and Technology, 7489 Trondheim, Norway, ^10^Research Institute IRCSS “Fondazione Santa Lucia”, Rome 00179, Italy, ^11^Molecular Connections Pvt. Ltd., Bangalore 560 004, India, ^12^Institut de Biologie et Chimie des Protéines, Unité Mixte de Recherche 5086, Centre National de la Recherche Scientifique, Université Lyon 1, Lyon, France and ^13^Structural and Computational Biology Unit, European Molecular Biology Laboratory (EMBL), Meyerhofstrasse 1, D-69117 Heidelberg, Germany

## Abstract

IntAct (freely available at http://www.ebi.ac.uk/intact) is an open-source, open data molecular interaction database populated by data either curated from the literature or from direct data depositions. IntAct has developed a sophisticated web-based curation tool, capable of supporting both IMEx- and MIMIx-level curation. This tool is now utilized by multiple additional curation teams, all of whom annotate data directly into the IntAct database. Members of the IntAct team supply appropriate levels of training, perform quality control on entries and take responsibility for long-term data maintenance. Recently, the MINT and IntAct databases decided to merge their separate efforts to make optimal use of limited developer resources and maximize the curation output. All data manually curated by the MINT curators have been moved into the IntAct database at EMBL-EBI and are merged with the existing IntAct dataset. Both IntAct and MINT are active contributors to the IMEx consortium (http://www.imexconsortium.org).

## INTRODUCTION

Experimental approaches to determine molecular interactions often require that interactions are determined in non-physiological conditions which can result in significant false positive and false negative rates. Knowledge of experimental details, such as the experimental technology used, cellular context, protein modifications and expression systems are essential to evaluate the reliability of an interaction report, or to combine multiple reports into an overall reliability assessment. Experiments often determine additional valuable biological information in addition to the identity of the interaction partners, for example, interacting domains or sites of post-translational modifications. Curating interactions to the required level of detail to capture this information requires both a relatively complex data structure and frequent updates, including the mapping of binding regions, point mutations and post-translational modification to a specified sequence within a reference protein sequence database. An update to a predictive gene model may result in a corresponding change to the protein sequence(s) derived from it. Interactions involving domains and/or residues of that protein sequence within an interaction database will then require a corresponding update to ensure the mapping to the updated sequence is correct. Update pipelines need to be run regularly, in line with the release cycle of the sequence database, namely every 4 weeks in the case of UniProtKB (1). Similarly, controlled vocabularies (CVs) used to annotate interaction data need to be refreshed with every new release. The systematic capture of detailed information requires both a complex underlying data model and a curator-friendly, efficient data capture system using web services to provide on-demand data import from external resources and frequently updated CVs. Such complex curation environments are much more expensive in terms of development and operational cost than the public facing, read-only website components of a typical molecular interaction database.

The IntAct molecular interaction database has existed since 2002 to serve richly curated molecular interaction data provided in community accepted standard formats to a broad user community (2). Data users range from cell biologists exploring the fine details of the mechanism by which a specific protein binds to protein partners to analysts utilizing the entire known interactome of a particular model organism to perform network analysis on the results of a large-scale ‘Omics experiment. The IntAct database is based on a relational database management system (Oracle; Postgres version available), Hibernate object-relational mapping, Java-based middleware, Lucene/SOLR indexing and a web-based front end for public search, visualization and download of the data. From its earliest implementations in 2002 onwards, the IntAct curation system has been developed as a web-based platform, to allow collaborative curation by physically remote partners, originally mainly a small group of UniProtKB/Swiss-Prot curators based in Geneva, Switzerland. While the IntAct data model is capable of describing interactions between any biomolecule type, the primary curation focus has always been on experimentally verified protein–protein interactions. IntAct curators capture this data from the literature, in a series of curation projects directed by grant funding and the requirements of collaborating experimental groups. In addition, the direct submission of experimental data as part of the publication process is actively encouraged by an increasing number of journals and is supported by the IntAct curation team. Any submitted data are kept confidential until the accompanying publication is released but formatted data files can be provided to the submitter directly from the editorial tool to ease input into tools such as Cytopscape (3). IntAct is released at least monthly, and all existing publications are available from the IntAct ftp site in PSI-MI XML and MITAB 2.5, 2.6 and 2.7 formats as well as via direct download from the website where data are additionally available in RDF and XGMML formats (2,4,5). Data can also be queried using the PSICQUIC web service, and IntAct IMEx data are also accessible via the IMEx website (6). All IntAct software, including the web-based curation tool, is free and open source, and can be used, modified and redistributed under the terms of the Apache Software License.

The MINT (Molecular INTeraction) database was created in 2002 at the University of Tor Vergata, Rome (7), independent of IntAct in funding and organization, but with very similar aims, curating experimentally derived protein interactions, with specific curation targets determined by the requirements of the large experimental activity of the group, for example, SH3 domain-based interactions (8). A major activity was and is the close collaboration with the *FEBS Letters Journal*, where MINT curates all protein interactions published in the journal (9,10). MINT developed its own software infrastructure for curation as well as data dissemination, but already in 2006 decided to adapt the IntAct relational database implementation to reduce the overhead of database development and maintenance. The MINT web interface remained distinct and based on an IntAct-independent code base.

Unlike protein structure or sequence databases, where single, collaborative database projects such as PDBe (11) and UniProt (1) provide the vast majority of public data, curation of molecular interaction data is mainly performed by a relatively large number of small to medium, independently funded projects (12). Often the driver for a curation effort is not general resource provision, but local expertise in a specific scientific domain, which is exploited for the curation of reference datasets, often in direct support of experimental activities in the same group. As an example, MatrixDB (Universite de Lyon, France) (13) is highly specialized in the curation of extracellular domain interactions. While domain-specific, expert-curated interaction datasets are likely to be of high quality and scientific relevance, this distributed approach also has significant risks. Recognizing the risks of data fragmentation, redundant curation and redundant software development, many leading interaction databases, including IntAct, MINT and DIP (14), have since 2002 (15) engaged in increasingly closer collaboration, moving from definition of common data representation [Human Proteome Organisation Proteomics Standards Initiative (HUPO PSI)-MI] (4) to co-ordinated curation strategies (IMEx) (6) and common computational query interfaces (PSICQUIC) (5). It is an essential part of the IMEx Consortium agreement that, should a member database lose funding or cease activities due to transfer of group interests, the data be passed to another IMEx member database for long-term data stewardship. As of January 2012, the MPIDB database (16) ceased active curation and since that date the IMEx records for that database are present within and updated by IntAct. These additional 363 publications significantly increased the IntAct support for microbial interactions.

## COMMUNITY ACCESS TO A WEB-BASED CURATION TOOL

As previously described (2), IntAct has invested heavily in the development of a sophisticated web-based editorial tool, enabling the systematic capture of the complexities of a molecular interaction experiment to either IMEx (6) or MIMIx-level (17) as determined by the curator. Part of the motivation for providing the entire IntAct code base as open source was to reduce redundant development of interaction database platforms. It was originally envisaged that individual groups would emulate the original MINT model and locally install versions of IntAct to curate and maintain their own interaction data. However, as described earlier, this still leaves such groups with a heavy data maintenance overhead to keep records synchronized with sequence database and CV updates. Over the last few years, several groups have therefore preferred to curate directly into IntAct and make use of the existing IntAct data maintenance pipeline. This is not ‘community annotation’ *per se*, as these all represent highly trained M.Sc. and Ph.D. level curators, with IntAct staff providing the appropriate level of training and also quality control of all records produced to ensure curation standards and data quality remain consistent across the entire dataset. When requested, IntAct makes interaction records available in specific download formats for each contributing group, and indeed for other databases wishing to display interaction data. For example, a number of UniProtKB/Swiss-Prot curators at both the Swiss Institute of Bioinformatics and EBI have for several years annotated molecular interactions directly into IntAct. IntAct then scores and filters all interaction evidences in the database and exports a high confidence subset, with a high degree of probability that the molecule pairs described physically interact with each other, back to UniProtKB (1), neXtProt (18) and the Gene Ontology annotation project (19) in a distinct format for each. Other databases such as I2D (Interologous Interaction Database) (20), which curates interaction protein interaction data relevant to the development of cancer and InnateDB (21), capturing both protein and gene interactions relevant to the process of innate immunity, choose to select appropriate records directly from the ftp site for subsequent import into their own resources. Both databases use the IntAct editor to perform IMEx-level curation (6). The contract curation company, Molecular Connections (www.molecularconnections.com/), perform pro bono public domain data curation via the IntAct database. AgBase, a curated resource of animal and plant gene products, captures data subsequently imported into their host–pathogen database (http://www.agbase.msstate.edu/hpi/main.html). The Cardiovascular Gene Ontology Annotation Initiative at University College London is building an interactome of cardiovascular associated proteins (http://www.ucl.ac.uk/cardiovasculargeneontology/).

Several of the groups contributing data to IntAct are interested in molecule types other than proteins. MatrixDB (13) database focuses on interactions established by both extracellular proteins and polysaccharides, and uses the IntAct curation tool to input interaction data which is subsequently downloaded and transferred into their own database environment. Similarly, a very new collaboration with the Norwegian University of Science and Technology, Trondheim is further extending the initial efforts of the IntAct curators into capturing transcription factor interactions with the genes to which they bind. To meet both these additional use cases, the IntAct editorial tool has been extended to facilitate access to both small molecule data from ChEBI (22) and information on the gene derived from Ensembl (23). In each case, web services enable key information to be downloaded from an appropriate reference database and stored internally within IntAct, enabling curators to annotate interaction data relevant to those entities. In the near future, the increasing amount of RNA-based interaction data will also present new challenges to the molecular interaction curation community and the development of reference resources such as RNAcentral (24) will be critical to the capture of these important interactomes.

Each group is provided with its own PSICQUIC web service (5) running from within the IntAct database so that each data provider can choose to embed its own web service within a web page or tool, completely independently of other data present in the database. IntAct has also made several changes to enable additional credit to be given to external contributors. In addition to minor cosmetic changes to the website, such as displaying the logos of these groups and also the source of each data entry in the database, a new Institute Manager facility has been added to the editorial tool, to enable each set of curators to be linked and statistics generated on request, to enable each institute to support internal and grant-driven data requirements.

**Figure 1.**
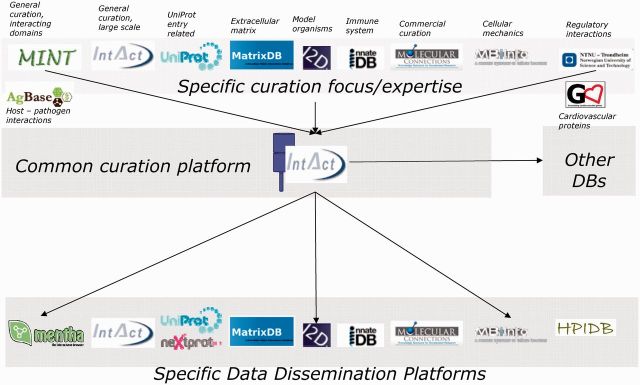
The web-based IntAct curation environment is used by currently 11 independent organizations, most of which re-export the data and present it through their own web interfaces.

The model of a shared curation environment and common data dissemination formats minimizes the development of redundant curation platforms and ensures compatibility of the curated data generated by all partners, while each partner can contribute their domain-specific curation expertise. On the other hand, the option of separate web interfaces for partners allows them to develop domain-specific websites for their communities, based on their own as well as other partners’ data (see Figure 1).

## THE MINTACT PROJECT

**Figure 2.**
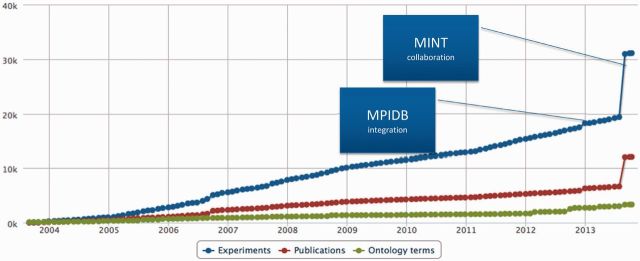
Growth of the IntAct database.

The use of IntAct as a shared curation platform has been boosted in Summer 2013, when IntAct and MINT joined forces to optimize curation and software development efforts. IntAct and MINT shared many commonalities and much of the basic maintenance and, as described earlier, update work to the underlying database infrastructure was largely redundant between the two groups. The two databases collaborated closely to work on the data formats and standards produced in collaboration with other members of the Molecular Interaction work group of the HUPO-PSI. This also included the development of curation standards and CV terms in addition to tools and services such as the PSICQUIC Web service. However, despite these shared efforts and their common infrastructure, both databases existed as physically separate entities and independently improved and updated their respective installations. It was therefore agreed, early in 2013, to merge this common effort, with a view to making optimal use of limited developer resources and maximize the curation output. To achieve this, the entire set of experimentally derived data manually curated from published literature was transferred from MINT to IntAct at EMBL-EBI. Locally maintained CVs such as the cell line and tissue lists used to describe the host organism in which experiments are undertaken, were manually aligned and merged into a single reference list. Centrally maintained CVs, for example, the PSI-MI CV were refreshed (4). Underlying protein sequences, and features mapped to them, were updated to the latest UniProtKB release before the two datasets were merged. Although the IMEx Consortium rules now prevent redundant curation of the same publication by multiple member databases, there were many examples of papers curated prior to 2006 which were present in both databases. In each case, the most richly annotated paper was selected as the dataset retained within the combined dataset. A new tagged data subset, Virus, was created to tag the manually curated papers from VirusMINT (25), and supplemented by additional papers containing virus–virus or virus–host interactions already present in the IntAct database. The final merged dataset (11 879 publications, 430 134 binary interactions) was released in August 2013, resulting in a huge increase in the data available. As detailed in Figure 2, the number of publications in IntAct almost doubled from 6600 to 12 000. While the joining of the two datasets was a huge effort for all involved curators and developers, it ultimately took only a little over 1 month from the original transfer of the MINT dataset end of August to the first joint release in early September. Taking into account the huge size of the MINT dataset, this is an impressive demonstration of the usefulness of common curation strategies and data representation between independent databases in the same or closely related domain.

As from June 2013, the MINT curators curate new publications directly into the IntAct database, and the input into ongoing MINT projects has been transferred to the IntAct platform. This includes the creation of the Structured Digital Abstracts for FEBS letters which are now generated by IntAct, with the agreement of the editorial board of that journal. The MENTHA interactome browser (26) will continue to be built on the PSICQUIC web services of IMex databases (6) and BioGRID (27), as before. The merger of curation activities gives users access to an improved and continually updated single dataset, and the European tax payer gets an improved return on investment as improvement to the underlying database structure and website development efforts need only be funded once, at a single site with both groups contributing to long term data maintenance efforts. Future software developments, such as upgrades to new versions of data formats, will be undertaken jointly by the two groups, maximizing resources in a time of restricted funding in Europe.

## FUTURE PLANS

Over the last few years, IntAct has focused very much on data input, developing a sophisticated curation tool, curation life cycle and working with many other groups to extend the effective coverage of the literature achieved by the database. While this will continue, it is intended in the immediate future to focus on the presentation of the resulting data to those members of the IntAct user community who access the data via the website—improving search, filtering and graphical capabilities. New ways to display data need to be explored to cope with the ever-growing volume available. It is estimated that the volume of data held by the IntAct database alone may grow to 750 000 binary interaction evidences in the next 5 years, and while this can be presented using current simplistic network views, it is almost impossible to derive useful information directly from the resulting graphic. Novel methods of merging data and displaying clustered by pathway, process or subcellular location have to be considered.

Interaction data are becoming ever-more sophisticated and new ways of visualizing data need to be developed to capture this. One example of this is the generation of dynamic interaction data, in which changes in protein complex composition in response to stimuli, be these biochemical (e.g. in response to increasing concentrations of an agonist such as a growth factor), temporal (such as different phases of the cell cycle) or environmental (dark versus light). IntAct has developed an extension of the CytoscapeWeb viewer (28) to present such dynamic changes as an animation driven by radio-buttons, provided data are entered into the database under appropriate annotation topic headings. The entire potential protein complex is displayed in the viewer, with those interactions relevant under a particular condition, or set of conditions, highlighted with the edge colour in red (http://www.ebi.ac.uk/intact/interaction/EBI-6263088). Future plans include extending this viewer, and indeed the underlying database model, to cope with concomitant changes in expressed protein level within the complex as improvements in mass spectrometry-based quantitative proteomics techniques means that such data become available.

We are constantly trying to improve our databases and services in terms of accuracy and representation and actively encourage user feedback. Please contact IntAct if you have any questions via http://www.ebi.ac.uk/support/index.php or email us directly at intact-help@ebi.ac.uk. Information about curation is provided at http://www.ebi.ac.uk/intact/pages/documentation/data_curation.xhtml and at http://www.ebi.ac.uk/intact/pages/documentation/data_submission.xhtml about data submissions. Extensive documentation and training material on how to best use our resource is available at http://www.ebi.ac.uk/training/networks. Curation groups interested in capturing interaction data who would like access to the editorial tool are encouraged to contact the IntAct molecular interaction database to discuss this further (intact-help@ebi.ac.uk).

## FUNDING

European Commission under PSIMEx, contract [FP7-HEALTH-2007-223411], SyBoSS [FP7-HEALTH-2009-242129] and Affinomics [FP7-241481]; IntAct is additionally supported by The Michael J. Fox Foundation for Parkinson's Research LRRK2 Biology LEAPS and NHLBI Proteomics Center Award [HHSN268201000035C]; MINT is supported by AIRC [10360], ERC [322749-DEPTH] and Telethon [GGP09243]. The Cardiovascular Gene Annotation Initiative is supported by British Heart Foundation grant [RG/13/5/30112]; SIB Swiss Institute of Bioinformatics receives financial support from the Swiss Federal Government through the State Secretariat for Education, Research and Innovation (SERI). Funding for open access charge: European Commission grant Affinomics [FP7-241481].

*Conflict of interest statement.* Arathi Raghunath is an employee of Molecular Connections, a contract annotation company.
